# Correlation between Anatomopathological Aspects and Pelvic Pain in Women with Deep Infiltrating Endometriosis

**DOI:** 10.1055/s-0043-1772473

**Published:** 2023-12-23

**Authors:** Daniela Angerame Yela, Mariana Sousa Sguerra Silva, Larissa Eloy, Cristina Laguna Benetti-Pinto

**Affiliations:** 1 School of Medicine, University of Campinas, Campinas – SP, Brazil.

**Keywords:** Deep infiltrating endometriosis, Histology, Pain, Endometriose profunda, Histologia, Dor

## Abstract

**Objective** To correlate the morphological aspects with pelvic pain in women with deep infiltrating endometriosis.

**Methods** A retrospective study with 67 women with deep endometriosis who underwent surgical treatment in a tertiary hospital from 2007 to 2017. The following variables were considered: age, parity, body mass index, site of involvement, hormonal treatment before surgery, pelvic pain, and morphometric analysis. The histological slides of the surgical specimens were revised and, using the ImageJ software for morphometric study, the percentages of stromal/glandular tissues were calculated in the histological sections.

**Results** The mean age of the women was 38.9 ± 6.5 years. The mean pain score was 8.8 ± 1.9 and the mean time of symptomatology was 4.7 ± 3.5 years, with 87% of the patients undergoing hormone treatment prior to surgery. The average expression of CD10, CK7, and S100 markers was 19.5 ± 11.8%, 9.4 ± 5.9%, and 7.9 ± 5.8% respectively. It was found that the greater the expression of CD10, the greater the level of pain ( *p* = 0.02). No correlation was observed between the expression of CD10, CK7, and S100 markers and age and duration of symptoms.

**Conclusion** Women with deep infiltrating endometriosis have a positive association between the level of pain and the fibrosis component in the endometrial tissue's histological composition.

## Resumo

**Objetivo** Correlacionar os aspectos morfológicos com a dor pélvica em mulheres com endometriose profunda.

**Métodos** Estudo retrospectivo com 67 mulheres com endometriose profunda submetidas a tratamento cirúrgico em hospital terciário de 2007 a 2017. As seguintes variáveis foram consideradas: idade, paridade, índice de massa corporal, local do acometimento, tratamento hormonal antes da cirurgia, dor pélvica e análise morfométrica. As lâminas histológicas das peças cirúrgicas foram revisadas e, por meio do software ImageJ para estudo morfométrico, foram calculadas as porcentagens de tecidos estromais/glandulares nos cortes histológicos.

**Resultados** A média etária das mulheres foi de 38,9 ± 6,5 anos. O escore de dor médio foi de 8,8 ± 1,9 e o tempo médio de sintomatologia foi de 4,7 ± 3,5 anos, sendo que 87% das pacientes realizavam tratamento hormonal antes da cirurgia. A expressão média dos marcadores CD10, CK7 e S100 foi de 19,5 ± 11,8%, 9,4 ± 5,9% e 7,9 ± 5,8%, respectivamente. Verificou-se que quanto maior a expressão de CD10, maior o nível de dor ( *p* = 0,02). Não foi observada correlação entre a expressão dos marcadores CD10, CK7 e S100 com a idade e duração dos sintomas.

**Conclusão** Mulheres com endometriose profunda apresentam associação positiva entre o nível de dor e o componente de fibrose na composição histológica do tecido endometrial.

## Introduction

Endometriosis can be conceptualized as the presence of ectopic endometrial tissue, gland and/or stroma outside the uterine cavity inducing a chronic inflammatory reaction. 1 It is a common gynecological pathology, affecting one in every ten women in the general population. 2 Endometriosis can be didactically classified as peritoneal, ovarian, and deep, the latter being defined as the presence of invasion by more than 5 mm of the peritoneal tissue. 3 4

The clinical picture of endometriosis is mainly determined by pain (dysmenorrhea, dyspareunia, chronic pelvic pain, dyschezia, and dysuria) and infertility. Endometriosis can cause pain through different mechanisms such as inflammation, pressure, adherence, neural involvement, psychological factors, and peritoneal prostaglandins. The pain can be continuous/intermittent, cyclical (related to the menstrual period), or acyclical. 5 6 7

The treatment of endometriosis can be clinical, surgical, or combined. The treatment indication must be individualized, and its choice depends on the woman's age, desire for pregnancy, severity of the symptoms, type and location of lesions, and the disease's stage. 3 6 Currently, one of the main surgical indications in the treatment of deep endometriosis is poor response to clinical treatment. 8

This broad spectrum of response to hormone therapy may be due to different cell populations found in endometriosis foci. It is believed that women who have foci with a greater glandular population in relation to the stromal and/or fibrotic may have a better response to hormone therapy in terms of pain, as well as women whose endometriosis foci have a greater number of estrogen receptors / progesterone. 9

There is also the possibility that different neural densities and inflammatory cytokines in endometriotic tissue are closely related to pain intensity. The ectopic endometriotic tissue would favor the production of cytokines, generating local inflammation and a greater proliferation of local neural fibers, which would intensify the pain symptoms. 9

Although the painful manifestation of endometriosis is a frequent complaint, compromising the lives of affected women, its relationship with the degree of endometriosis and the different types of injury has not yet been fully elucidated. Studies correlating the different types of injury, percentage of different types of cells present, and their relationship with drug response may aid the choice of therapeutic options available. Thus, this study aims to correlate morphological aspects with pelvic pain in women with deep infiltrating endometriosis undergoing surgical treatment.

## Methods

This is a retrospective study of 67 women with deep infiltrating endometriosis who underwent surgical treatment at a tertiary hospital from 2007 to 2017. The women were identified through the electronic medical record system. Of the 876 women followed up during this period, only 70 were referred for surgical treatment, and 67 were included in the study. The surgical blocks were then located in the Department of Pathological Anatomy. The block was selected by reviewing the slides stained with hematoxylin and eosin from the surgical specimens, and those with highest percentages of endometriotic cells were chosen.

The areas corresponding to the stromal and glandular components of the endometriosis lesions were measured, and the respective percentages were calculated for each one. For better identification of these components, markers were used. The markers used were CD10 and S100 to identify stromal cells and cytokeratin 7 (CK7) to identify the glandular component.

The results of the histological analysis with the percentages of stromal/glandular tissues were related to the following clinical data: age, parity, body mass index (BMI, calculated based on weight in kilograms divided by the square of height in meters), site of endometriosis involvement, use of medication before surgery (combined oral contraceptives or progestins), pelvic pain with intensity being graded from 0 to 10 according to the visual analogue scale (VAS), and time of pain symptom. Data were collected by the researchers responsible through the information contained in medical records.

This study was approved by the Ethics and Research Committee of the University of Campinas, Brazil, under the number CAAE 34929120.4.0000.5404.

Immunohistochemical reactions for CD10, S100, and CK7 were performed at the Laboratory of Immunohistochemistry of the hospital.

The materials, previously fixed in 10% formalin and embedded in paraffin, were submitted to histological sections of 4 micrometers of thickness, which were placed on signed slides, after which they were deparaffinized with 3 xylol baths at room temperature. Afterwards, the slides were bathed in absolute alcohols three times, once in 80% alcohol and once in 50% alcohol for progressive hydration. They were then washed in running water for 3 minutes and, finally, rinsed in distilled water. In order to inhibit endogenous peroxidase, the slides were bathed for 5 minutes each in a hydrogen peroxide solution at room temperature, then washed again in running water for 3 minutes and then rinsed in distilled water.

Antigenic retrieval was performed by immersing the slides in 0.05M Tris-EDTA pH 8.9 buffer for 30 minutes at approximately 95°C in a steam pan, with subsequent cooling for 15 minutes for anti-CK7 monoclonal mouse antibodies (clone OV-TL12/30, DAKO, Glostrup, Denmark), monoclonal mouse anti-CD10 (clone 56C6, DAKO) and polyclonal rabbit anti-S100 (DAKO), all with dilution 1:100. Afterwards, they were washed in running water and distilled water.

The primary antibodies were dripped onto the respective histological preparations at the aforementioned dilutions, and the slides were incubated in an oven at 37°C for 30 minutes. Afterwards, they were incubated for 16 to 20 hours (overnight) at 4°C in a humid chamber.

Subsequently, they were washed three times in phosphate buffer saline (PBS) pH 7.4 solution at room temperature. The slides were then incubated for one hour at 37°C with a cocktail of polymers labeled with peroxidase, using the advanced detection system (DAKO), and then immersed again in PBS.

Staining was performed with the diamino-benzidine (DAB) brown chromogen kit (DAKO, REF – 3468) for 5 minutes at 37°C. The material was washed in running water, counterstained with the Mayer hematoxylin, dehydrated (three baths in ethyl alcohol and three baths in xylene) and the coverslips were glued with the Entellan resin (ProSciTech Pty. Ltd. Kirwan, QLD, Australia).

In addition to the cases in the study group, a case of liver in the cirrhosis phase was selected to control the immunohistochemical reactions for the three markers, for which the positivity of the reaction is considered: CD10–membrane expression in the bile canaliculi of the hepatocytes; CK7–cytoplasmic expression in bile duct epithelium; and S100–cytoplasmic expression in neural plexuses.

After this process, the slides were photographed in a Zeiss Axiophot 2 microscope (Carl Zeiss Meditec, Jena, Thuringia, Germany), with an Olympus DP72 camera (Evident Corp., Shinjuku-ku, Tokyo, Japan) using the cellSens Standard (Evident Corp.) software that processes the images for the computer. Each slide was photographed in three different regions, where markers were expressed in greater proportions. The histological analysis was performed with the help of a pathologist. After being photographed, each image was inserted into the ImageJ (LOCI, University of Wisconsin, WI, USA) software for morphometric study, which measures the percentage of expression of the analyzed markers. 10

For morphological evaluation, a histological classification was applied comprising four different forms of endometriosis, based on the degree of differentiation. 11 12 13 The first is a well-differentiated glandular form, with the presence of surface epithelium or epithelium with glandular to cystic formations; the cells are indistinguishable from a normal endometrium during different phases of the menstrual cycle (proliferative/follicular, secretory/luteal, menstrual, and regenerative). The second is a pure stromal form of endometriosis, without any surface or glandular epithelium; the stroma also closely resembles that of the normal endometrium during different phases of the menstrual cycle. The third is a glandular pattern of mixed differentiation, with the epithelium being composed of cylindrical to columnar endometrial cells, cuboidal to low flattened cells, undifferentiated cells, and, sometimes, cells with other Mullerian histological patterns (serous or mucinous cells). The fourth is a poorly differentiated form that is characterized by a uniquely undifferentiated glandular pattern in which the surface epithelium or glandular/cystic formations are lined exclusively by low to flat cuboidal, mesothelial cells, or appear as small epithelial nests or islands.

The variables were described as frequency, mean, and standard deviation (SD). The Chi-square and Fisher exact tests were used to detect associations between categorical variables. The Kruskal-Wallis and Mann-Whitney tests were used to detect the association between continuous variables. The Spearman correlation coefficient was used to analyze the relationships between numerical variables. A probability value ( *p* -value) of <0.05 was considered statistically significant. The SAS software (SAS Inc., Cary, NC, USA) version 9.04 was used for all statistical analyses.

## Results

The mean age of the women was 38.9 ± 6.5 years and the mean BMI was 25.8 ± 4.5 kg/m2. The average pain level was 8.8 ± 1.9, and the average time of symptom onset was 4.7 ± 3.5 years. Among the 67 women, 55.2% were nulliparous and 87% underwent hormone treatment prior to surgery. The sites of disease involvement were: rectosigmoid (65.6%), ovary (43.2%), peritoneum (16.4%), ureter (0.5%), and uterosacral (0.1%) ( Table 1 ).

**Table 1:** Clinical characteristics of women with deep infiltrating endometriosis undergoing surgical treatment (n = 67)

Variables	Mean ± SD / n(%)
Age (years)	38.9 ± 6.5
BMI (kg/m ^2^ )	25.8 ± 4.5
Nulliparous	37(55.2)
Pelvic pain	8.8 ± 1.9
VAS (7–10)	60(89.5)
VAS (4–6)	7(10.5)
Hormonal treatment	58(86.5)
Pain time (years)	4.7 ± 3.5

**Abbreviations:** SD, standard deviation; BMI, body mass index; VAS, visual analog pain scale.

The average expression of anatomopathological markers CD10, CK7, and S100 was 19.5 ± 11.8%, 9.4 ± 5.9%, and 7.9 ± 5.8%, respectively. Regarding the histological classification, 80% of the slides showed an undifferentiated glandular tissue pattern. There was no significant difference between the percentage of CD10, CK7, and S100 markers and the site of involvement of endometriosis in women ( *p* = 0.37, 0.12, and 0.09, respectively). No difference was observed between expression of CD10, CK7, and S100 markers and hormone treatment ( *p* = 0.79, 0.83, and 0.74, respectively). Women who did not undergo hormone treatment had a mean CD10 expression of 20.2 ± 10.6%; of 8.4 ± 5.0% for CK7; and 8.0 ± 7.5% for S100, while those who underwent hormonal treatment had an average of CD10 of 19.4 ± 12.0; 9.5 ± 6.1 for CK7; and 7.9 ± 5.6 for S100 ( Table 2 ).

**Table 2:** Evaluation of the percentage of anatomopathological markers CD10, CK7 and S100 according to the site of involvement of endometriosis and hormonal treatment in women (n = 67)

	Ovary	Peritoneum	Rectosigmoid	*p* *	Treatment	Without treatment	*p* **
CD10 (%)	17.6 ± 13.8	15.6 ± 12.7	17.5 ± 11.0	0.37	19.4 ± 12	20.2 ± 10.6	0.79
CK7 (%)	10.7 ± 8.6	8.3 ± 4.4	8.7 ± 5.9	0.12	9.5 ± 6.1	8.4 ± 5.0	0.83
S100 (%)	9.3 ± 8.3	6.4 ± 2.9	6.1 ± 4.2	0.09	7.9 ± 5.6	8.0 ± 7.5	0.74

**Notes:** *Kruskal-Wallis test. **Mann-Whitney test.

It was found that a greater the expression of CD10 correlated with greater levels of pain ( *p* = 0.02). A positive correlation was also found between the expression of S100 and the BMI ( *p* = 0.002). No correlation was observed between the expression of CD10, CK7, and S100 markers and age or duration of symptoms ( Table 3 ).

**Table 3:** Correlations between age, pain level, duration of symptoms and body mass index with expression of markers CD10, CK7 and S100 (n = 67)

		Age	Pain score	Pain time	BMI
CD10	r	0.069	0.271	0.108	0.054
	*p*	0.57	0.02	0.38	0.66
CK7	r	0.021	−0.022	−0.106	−0.100
	*p*	0.85	0.85	0.39	0.41
S100	r	−0.008	0.080	0.166	0.359
	*p*	0.94	0.51	0.17	0.002

**Abbreviation:** BMI, body mass index. **Note:** r = Spearman correlation coefficient.

## Discussion

This study observed a positive correlation between the level of pain and the percentage of CD10 (stromal marker). There was no significant difference between marker expression and the site of endometriosis involvement and treatment performance. There was no correlation between marker expression and age or duration of symptoms.

Most of the women in our study had a histological pattern classified as undifferentiated glandular. Studies in the literature indicate that women with deep infiltrating endometriosis present this undifferentiated pattern. 11 12 Additionally, studies point out that women who present the undifferentiated glandular pattern have the worse response to hormonal treatment. 11 12 In our study, most women underwent surgical treatment due to poor response to hormone treatment—the mean pain score was nine, which can be explained by the undifferentiated histological pattern of the lesions.

Histological analysis of deep infiltrating endometriosis often shows undifferentiated glandular and/or stromal cells surrounded by a significant amount of fibrotic tissue. 13 14 Endometriosis-related fibrosis represents a complex phenomenon, with underlying mechanisms that are still unclear. Fibrosis is consistently present in all forms of this disease and contributes to the classic symptoms of pain and infertility related to endometriosis. 9 The main component of nodular lesions is not endometrial tissue, but fibromuscular tissue with sparse finger-like extensions of glandular tissue and stroma. 15

Our results showed higher levels of fibrosis in the evaluated biopsies (higher expression of CD10). Therefore, this finding suggests that hormone treatment would not be effective for the fibrotic portion of the disease. Pain mechanisms, especially in women who did not respond to hormone treatment, are complex and could also be related to other multifactorial elements such as inflammation, oxidative stress, and genetics. 16

There are several mechanisms that contribute to chronic pain in endometriosis. Among them are neurogenic inflammation, neuroangiogenesis, peripheral sensitization, and central sensitization. As women with endometriosis may also have other comorbid conditions such as irritable bowel syndrome and overactive bladder syndrome, the study by Maddern et al. highlights how the common nerve pathways that innervate the colon, bladder, and female reproductive tract may contribute to pain through crossorgan sensitization. 17

Endometriosis is a complex disease characterized by a relevant component of fibrosis and adhesions. The identification of specific histopathological characteristics remains extremely important for the diagnosis of endometriosis. 18 This study establishes a relationship between the level of pain and the highest expression of the CD10 marker, which stains stromal cells. Thus, it corroborates the prerogative that women with more pain would have more fibrosis in relation to glandular cells and, therefore, would not respond to hormonal treatment.

Current therapeutic options provide pain relief for over 6 months in only 40 to 70% of women. 19 20 As such, a greater understanding of the mechanisms underlying endometriosis-induced pain is needed to achieve better clinical outcomes in the future. 17

This study has limitations, such as a small sample size and the absence of a control group. Further studies and the evaluation of a larger number of cases are needed to establish the relationship between fibrosis and the degree of clinical response. In this way, we intend a greater understanding of the chronic pain associated with endometriosis and the identification of possible targets for pain control that can help improve the quality of life of people who suffer from this disease.

## Conclusion

Women with deep infiltrating endometriosis have a positive association between the morphometric aspects of endometriotic lesions (CD10 marker) and pain.
